# Periodic Analysis of Surface Acoustic Wave Resonator with Dimensionally Reduced PDE Model Using COMSOL Code

**DOI:** 10.3390/mi12020141

**Published:** 2021-01-28

**Authors:** Qiaozhen Zhang, Zhenglin Chen, Yanguang Chen, Jiahe Dong, Panliang Tang, Sulei Fu, Haodong Wu, Jinyi Ma, Xiangyong Zhao

**Affiliations:** 1 College of Information, Mechanical and Electrical Engineering, Shanghai Normal University, Shanghai 200234, China; zhangqz@shnu.edu.cn; 2 China Electronics Technology Group Corporation No.26 Research Institute (SIPAT), Chongqing 400060, China; yanguangchen88@163.com (Y.C.); jiahedongpaper@163.com (J.D.); PanTangLD@163.com (P.T.); JYMSPE1970@163.com (J.M.); 3 Key Laboratory of Advanced Materials (MOE), School of Materials Science and Engineering, Tsinghua University, Beijing 100084, China; fusulei@mail.tsinghua.edu.cn; 4 Key Laboratory of Modern Acoustics, Ministry of Education, Department of Acoustic Science and Engineering, School of Physics, Nanjing University, Nanjing 210093, China; haodongwu@163.com

**Keywords:** SAW resonator, partial differential equations, finite element method, COMSOL multiphysics

## Abstract

Radio-frequency (RF) surface acoustic wave (SAW) resonators used as filters and duplexers are mass-produced and widely used in current mobile phones. With the numerous emergences of the diverse device structure, a universal method used for the accurate and fast simulation of the SAW resonator calls for urgent demand. However, there are too many instances where the behavior of the entire acoustic resonator cannot be characterized rapidly and efficiently due to limitations in the current computer memory and speed. This is especially true for SAW resonators configured with long arrays of inter-digital transducers (IDTs), and we have to resort to a periodic analysis. In this paper, the previously reported generalized partial differential equations (PDE) based on the two-dimensional finite element method (2D-FEM) model is extended to analysis for the periodic structure of the SAW resonator. We present model order reduction (MOR) techniques based on FEM and periodic boundary conditions to achieve a dimensionally reduced PDE model without decreasing the accuracy of computations. Examples of different SAW devices, including the regular SAW, IHP-SAW and TC-SAW resonators, are provided which shows the results of the periodic analysis compared with the experimental results of the actual resonators. The investigation results demonstrate the properties of the proposed methodology and prove its effectiveness and accuracy.

## 1. Introduction

Surface acoustic wave (SAW) devices are used as resonators in electronic systems in a wide range of applications: mobile communications [1,2], sensors [3,4] and actuators [5,6], etc. Because of their excellent performance, including low insertion loss, high isolation, etc., filters and duplexers based on radio-frequency (RF) SAW resonators are mass-produced and widely used in current mobile phones [7,8,9,10]. With the coming of the 5G era, more and more stringent requirements are imposed on the performance of the SAW devices. This promotes the emergence of a large number of new-type SAW devices, such as temperature-compensated SAW (TC-SAW) devices [11,12,13], incredible high-performance SAW (I.H.P. SAW) devices [14,15], and laterally-excited bulk-wave resonators (XBARs) [16,17]. Performances of those RF SAW devices are dominantly limited by their resonators. In order to meet the increasing demand for high-performance SAW devices, a new-type resonator with a more complicated structure and multilayer piezoelectric substrate has been continuously emerged [18,19,20]. Thus, SAW engineers are always seeking solutions to obtain more universal, fast and precise simulation tools used for characterizing the SAW resonator.

Currently, the detailed information of the resonator can be obtained by accurate calculation of finite-length resonator using finite element method/boundary element method FEM/BEM [21,22,23,24] or FEM-based hierarchical cascading technique (HCT) [25,26,27]. However, there are too many instances where the behavior of the finite-length resonator cannot be characterized rapidly and efficiently due to limitations in current computer memory and speed, even if the latest GPU-assisted HCT is performed [28]. Although true SAW resonators are configured with long arrays of inter-digital transducers (IDTs), substantial analytical and design information on the frequency characteristics of the acoustic resonator itself are essentially periodic. Thus, many researchers focus on the finite element analysis of periodic structures, which give rise to a significant reduction in the size of the numerical model and yield useful information for the whole resonator [29,30,31,32]. Among them, the periodic analysis based on FEM/BEM provided an effective way to accurately simulate the useful information of a normal SAW resonator [33,34,35]. Nevertheless, this method requires a long time to compute the Green function, especially for the multi-layered and intricate structure of SAW resonator, the Green function calculation in BEM becomes complex or even impractical. In contrast to FEM/BEM, pure FEM is generally effective and quite flexible: it allows one to handle arbitrary materials cuts, electrode shapes and multi-layered substrate, etc. [36,37]. Therefore, the pure FEM method is widely used to study the behavior of resonators even with more complicated structures and multilayer piezoelectric substrates. Yong et al. [30,38,39] proposed 2D-FEM and 3D-FEM models to achieve the periodic analysis of SAW resonators using FEM programming. As for FEM calculation, an adequate number of mesh grid (proportionate to degrees of freedom (DOFs)) is required to ensure the accuracy of SAW simulation. However, the more model’s DOFs the more computing resources are required. Yong’s FEM-based periodic analysis only performed DOFs eliminating located on sides of single-finger by using the anti-periodicity boundary conditions, and hence, the remaining part DOFs required to be calculated are still time-consuming. Due to the convenience and visualization of commercial FEM software COMSOL, the built-in quasi-3D FEM model based on COMSOL has drawn great attention [24,40]. To simplify and ensure accuracy, a continuous boundary condition is applied to the two interfaces of the 3D-block in the aperture direction to describe the infinite length of the aperture. In our previous work [41], the authors also proposed a similar partial differential equation (PDE)-based 2D-FEM model, which is theoretically equivalent to the built-in quasi-3D FEM model reported previously. Since the computation domain of the PDE-based 2D-FEM model is a 2D plane while that of the quasi-3D FEM model reported previously is a 3D block, the proposed PDE-based 2D-FEM model radically reduces the number of DOFs, and thus advantages in calculation speed.

In this work, the PDE-based 2D-FEM model is extended to the application for periodic analysis of SAW resonator. This paper presents model order reduction (MOR) techniques [42] based on FEM and periodic boundary conditions to achieve a dimensionally reduced PDE model without decreasing the accuracy of computations. First, the PDE-based 2D-FEM model for the half-period single-finger structure of the SAW resonator is solved by a solver of the PDE module embedded in COMSOL software. Then the FEM matrices containing all DOFs derived from COMSOL were transferred to MATLAB via LiveLink of COMSOL with MATLAB for the following MOR calculation. We investigate two significant contributions to achieving the dimensionally reduced model. On the one hand, by the order reduction technique of FEM, the dimensionality of the derived system equation can be largely reduced by eliminating the internal DOFs that are not of concern. On the other hand, considering the antiperiodic boundary conditions imposed on the left- and the right-hand edge of single-finger structure, the reduced system equation can be dimensionally reduced again by eliminating the DOFs located on left edge or right edge. Thus, the final system equation for characterizing the single-finger of resonator needs only DOFs located on one of the two sides and the potential scalar located on the surface of the electrode. This means the DOFs for the same model are reduced by nearly a third, and thus the calculation speed can be significantly accelerated under the condition of ensuring accuracy. 

The paper is organized as follows. Section 2 outlines the general procedure involved in the whole computation process and elaborates modelling of the reduced PDE model for periodic analysis. Section 3 demonstrates and validates the numerical simulation for three different mainstream commercial SAW devices, including the regular SAW resonator, IHP-SAW resonator and TC-SAW resonator, based on the proposed dimensionally reduced PDE model. Finally, conclusions are discussed in detail in Section 4.

## 2. Methodology

### 2.1. COMSOL Multiphysics and Computation Procedure

The COMSOL multiphysics software is a finite element tool for multi-physical coupling analysis in engineering applications and scientific research [43,44]. The built-in PDE module of COMSOL Multiphysics software provides a general form PDE interface for solving PDEs of the 2D-FEM model for the SAW device. Its PDEs are related to the conservation laws that govern many areas of physics, and the multiple dependent variables in PDEs can be coupled. Considering its modifiability of PDEs itself, the proposed method can be extended to the application for accurate analysis of SAW devices with more complicated cases. Moreover, COMSOL software can transform the PDE-based FEM model to the COMSOL code, which is flexible and feasible for users to modeling, mesh generation and properties definition. In our previous work, recently presented in *Micromachines* [41], a PDE-based 2D-FEM model is proposed and validated for SAW devices simulation. Considering its generality, this work extends the model to the periodic analysis of SAW resonator, and we investigate two significant contributions to achieving the dimensionally reduced PDE model with the help of COMSOL code. Additionally, the adaptive mesh refinement algorithm embedded in COMSOL is powerful to refine the meshes around the surface of the piezoelectric substrate, thus the solution of the SAW resonator is a fast convergence solution. Therefore, the proposed FEM model for analysis SAW resonator with complex structure can be completed effectively and efficiently. 

The general procedure involved in the whole computation process mainly divides into four steps. First, the PDEs-based of 2D-FEM model for the periodic structure of the SAW resonator is derived by rigorous derivation. The second step is that the geometry and mesh of the model are generated and PDEs of each region in the eigenvalue mode are written by COMSOL code. The third step is that the PDEs are solved by the solver of the PDE module embedded in COMSOL software, the derived FEM matrices from COMSOL were transferred to MATLAB via LiveLink for the remaining calculation. The last step is that MOR techniques based on FEM and periodic boundary conditions are employed to achieve a dimensionally reduced PDE model without decreasing the accuracy of computations. Based on COMSOL Multiphysics 5.5 and MATLAB 2020a, the calculation was implemented on two high-performance platforms equipped with two CPUs (Intel Xeon Gold 6226R CPU @ 2.90 GHz, 256 GB RAM) and two GPUs (NVIDIA Quadro GV100 GPUs, 32 GB HBM2 memory each, CUDA Compute Capability 10.0 and total of 5120 CUDA cores).

### 2.2. Reduced PDE Modelling for Periodic Analysis

A SAW resonator is an acoustic device with the frequency characteristic of standing acoustic waves in the substrate and electrodes, and thus complete mathematical modelling must consider all the practical factors occurring in the device substrate, such as the propagation loss, dielectric loss, electrode resistance loss and electrode shape, which makes it considering practical factors as much as possible. As mentioned above, the PDE model based on COMSOL is proposed to realize a practical 2D-FEM analysis of the SAW resonator. The use of COMSOL code performs to build modeling and solve equations numerically to obtain system solutions. Combining the advantages of the two can achieve high versatility.

#### 2.2.1. PDE-Based 2D-FEM Model

In our previous work [41], the constitutive equations of the piezoelectric film are derived detailly which can describe the behavior of SAW devices with arbitrary materials and structure. For convenience, we only restate the main derivation of the PDE-based model in piezoelectric devices [45].

(1) $${Τ}_{ij}=c_{ijkl}S_{kl}-e_{kij}E_{k}$$

(2) $$D_{i}=e_{ikl}S_{kl}+\varepsilon_{ik}E_{k}$$

where ${Τ}_{ij}$ and $S_{kl}$ are stress and strain tensors, respectively. $c_{ijkl}$, $e_{ikl}$ and $\varepsilon_{ik}$ is stiffness constant, piezoelectric stress constant and dielectric permittivity constant, respectively. $D_{i}$ and $E_{k}$ are the electric displacement vector and electric field, respectively.

And the relation of strain and mechanical displacement in the piezoelectric substrate can be written

(3) $$S=\nabla_{s}^{\prime }u$$

where

(4) $$\nabla_{s}=\left[\begin{array}{cccccc} \frac{\partial }{\partial x_{1}} & 0 & 0 & 0 & \frac{\partial }{\partial x_{3}} & \frac{\partial }{\partial x_{2}}\\ 0 & \frac{\partial }{\partial x_{2}} & 0 & \frac{\partial }{\partial x_{3}} & 0 & \frac{\partial }{\partial x_{1}}\\ 0 & 0 & \frac{\partial }{\partial x_{3}} & \frac{\partial }{\partial x_{2}} & \frac{\partial }{\partial x_{1}} & 0 \end{array}\right]$$

According to electrostatics, the relation among the electric displacement $D_{i}$, electric field $E_{k}$, electric potential $\phi_{k}$ are expressed as

(5) D=εE

(6) E=−∇ϕ

where the Nabla operator $\nabla =\left[\begin{array}{ccc} \frac{\partial }{\partial x_{1}} & \frac{\partial }{\partial x_{2}} & \frac{\partial }{\partial x_{3}} \end{array}\right]$.

**Figure 1:**
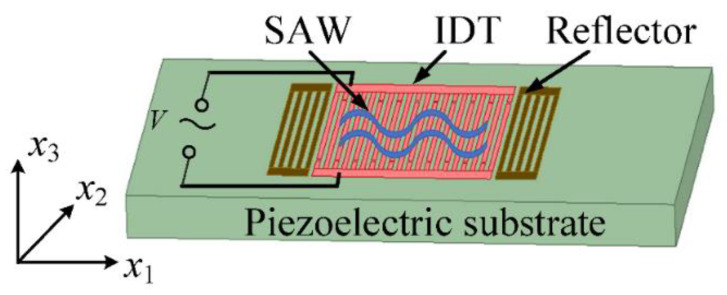
The schematic of a three-dimensional geometrical structure for a typical one-port surface acoustic wave (SAW) resonator (not to scale).

According to Newton’s Law and Maxwell’s equations, if there is no external force applied, the equilibrium equation for characterizing the full-scale SAW devices (seen in Figure 1) can be described with tensor form as Equations (7) and (8).

(7) $$\nabla_{s}c\nabla_{s}^{\prime }u+\nabla_{s}e^{\prime }\nabla \phi =\rho \ddot{u}$$

(8) $$-\nabla \cdot \varepsilon \nabla \phi +\nabla \cdot e\nabla_{s}^{\prime }u=0$$

where ρ is density.

The equilibrium Equations (7) and (8) are the three-dimensional (3D) differential equations for describing the full-scale SAW devices. For characterizing the SAW resonator as shown in Figure 1, the length of the aperture along $x_{2}$ direction is normally hundreds of times the IDT period. In this work, we start from the perspective of solving PDE Equations (7) and (8), the length of the aperture along $x_{2}$ direction is also assumed to be infinite (∂/∂$x_{2}$ = 0). Thus the operator $\nabla_{s}$ and Nabla operator ∇ can be expressed as following Equations (9) and (10), only the $x_{1}$ and $x_{3}$ coordinate axes are contained in the operator $\nabla_{s}$ and Laplace operator ∇.

(9) $$\nabla_{s}=\left[\begin{array}{cccccc} \frac{\partial }{\partial x_{1}} & 0 & 0 & 0 & \frac{\partial }{\partial x_{3}} & 0\\ 0 & 0 & 0 & \frac{\partial }{\partial x_{3}} & 0 & \frac{\partial }{\partial x_{1}}\\ 0 & 0 & \frac{\partial }{\partial x_{3}} & 0 & \frac{\partial }{\partial x_{1}} & 0 \end{array}\right]$$

(10) $$\nabla =\left[\begin{array}{ccc} \frac{\partial }{\partial x_{1}} & 0 & \frac{\partial }{\partial x_{3}} \end{array}\right]$$

**Figure 2:**
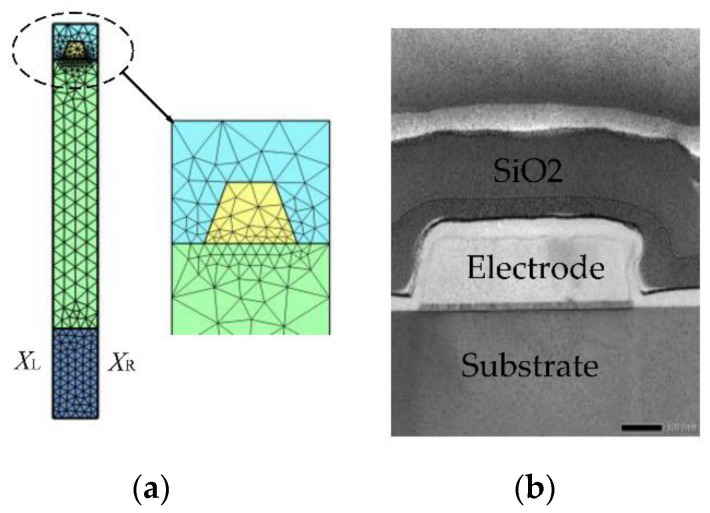
The single-finger SAW devices structure with trapezoid shape electrode, (**a**) 2D-FEM model, (**b**) cross-sectional scanning electron microscopy image of the actual device.

In this sense, the computation domain of the PDE-based 2D-FEM model is a 2D plane, as shown in Figure 2a. Therefore, all of the four solutions $u_{1},u_{2},u_{3}$ and ϕ of the equilibrium equation Equations (7) and (8) can be obtained by solving the following Equation (13) based on the 2D-FEM model. In addition, the trapezoid electrode was considered in the 2D-FEM model according to the actual process level, seen in Figure 2b.

Due to FEM software, COMSOL Multiphysics provides a mathematics module including different PDE module for equation-based modelling. A general form PDE interface is applied to solve Equations (7) and (8). In the case of four dependent variables $u_{1}$, $u_{2}$, $u_{3}$, ϕ, a general form system of the equation takes the following form (11) [46].

(11) $$e_{a}^{lk}\frac{\partial^{2}u_{k}}{\partial t^{2}}-\nabla \cdot c_{a}^{lk}\nabla u_{k}=f_{l}\;(\mathrm{in}\Omega )$$

where the equation index *l* and *k* ranges from 1 to 4, $e_{a}^{lk}$ is the mass coefficients, $c_{a}^{lk}$ is the diffusion coefficients, $f_{l}$ is the source term, Ω is the meshed domain used for FEM computational as shown in Figure 2a. Note that $e_{a}^{lk}$ and $c_{a}^{lk}$ are 4-by-4 matrices, $f_{l}$ are 4-by-1 matrices.

According to Equation (11), the equilibrium Equations (7) and (8) are converted to the COMSOL’s coefficient form. For simplicity, the long and tedious deduction of the COMSOL’s coefficient for PDE in this section is abridged. Instead, the results of these manipulations are presented, as follows

(12) $$\left[\begin{array}{cccc} \rho \omega^{2}u_{1} & & & \\ & \rho \omega^{2}u_{2} & & \\ & & \rho \omega^{2}u_{3} & \\ & & & 0 \end{array}\right]-\nabla \cdot \;\left[\begin{array}{cccc} \left[\begin{array}{cc} C_{11} & C_{15}\\ C_{51} & C_{55} \end{array}\right] & \left[\begin{array}{cc} C_{16} & C_{14}\\ C_{56} & C_{54} \end{array}\right] & \left[\begin{array}{cc} C_{15} & C_{13}\\ C_{55} & C_{53} \end{array}\right] & \left[\begin{array}{cc} e_{11} & e_{31}\\ e_{15} & e_{35} \end{array}\right]\\ \left[\begin{array}{cc} C_{61} & C_{65}\\ C_{41} & C_{45} \end{array}\right] & \left[\begin{array}{cc} C_{66} & C_{64}\\ C_{46} & C_{44} \end{array}\right] & \left[\begin{array}{cc} C_{65} & C_{63}\\ C_{45} & C_{43} \end{array}\right] & \left[\begin{array}{cc} e_{16} & e_{36}\\ e_{14} & e_{34} \end{array}\right]\\ \left[\begin{array}{cc} C_{51} & C_{55}\\ C_{31} & C_{35} \end{array}\right] & \left[\begin{array}{cc} C_{56} & C_{54}\\ C_{36} & C_{34} \end{array}\right] & \left[\begin{array}{cc} C_{55} & C_{53}\\ C_{35} & C_{33} \end{array}\right] & \left[\begin{array}{cc} e_{15} & e_{35}\\ e_{13} & e_{33} \end{array}\right]\\ \left[\begin{array}{cc} e_{11} & e_{15}\\ e_{31} & e_{35} \end{array}\right] & \left[\begin{array}{cc} e_{16} & e_{14}\\ e_{36} & e_{34} \end{array}\right] & \left[\begin{array}{cc} e_{15} & e_{13}\\ e_{35} & e_{33} \end{array}\right] & \left[\begin{array}{cc} -\varepsilon_{11} & -\varepsilon_{13}\\ -\varepsilon_{31} & -\varepsilon_{33} \end{array}\right] \end{array}\right]\left[\begin{array}{c} \nabla u_{1}\\ \nabla u_{2}\\ \nabla u_{3}\\ \nabla \phi \end{array}\right]=0$$

where ω is angular frequency.

#### 2.2.2. MOR Based on FEM

As mentioned above, the operator $\nabla_{s}$ and Nabla operator ∇ only include the $x_{1}$ and $x_{3}$ coordinate axes, and the derived PDE-based 2D-FEM model is theoretically equivalent to the pure quasi-3D FEM model previously reported [14,20,36,47]. The computation domain of the PDE-based 2D-FEM model is a 2D plane while that of the quasi-3D FEM model is a 3D block. This means the proposed PDE-based 2D-FEM model radically reduces the number of DOFs, and thus advantages in calculation speed.

Equation (12) is solved by the solver of the PDE module embedded in COMSOL Multiphysics, the FEM discretization results in the following matrix equation

(13) $$\left[\begin{array}{cccc} K_{\mathrm{LL}} & K_{\mathrm{LI}} & K_{\mathrm{LR}} & K_{\mathrm{LV}}\\ K_{\mathrm{IL}} & K_{\mathrm{II}} & K_{\mathrm{IR}} & K_{\mathrm{IV}}\\ K_{\mathrm{RL}} & K_{\mathrm{RI}} & K_{\mathrm{RR}} & K_{\mathrm{RV}}\\ K_{\mathrm{VL}} & K_{\mathrm{VI}} & K_{\mathrm{VR}} & K_{\mathrm{VV}} \end{array}\right]\left[\begin{array}{c} X_{L}\\ X_{I}\\ X_{R}\\ v \end{array}\right]-\omega^{2}\left[\begin{array}{cccc} M_{\mathrm{LL}} & M_{\mathrm{LI}} & M_{\mathrm{LR}} & M_{\mathrm{LV}}\\ M_{\mathrm{IL}} & M_{\mathrm{II}} & M_{\mathrm{IR}} & M_{\mathrm{IV}}\\ M_{\mathrm{RL}} & M_{\mathrm{RI}} & M_{\mathrm{RR}} & M_{\mathrm{RV}}\\ M_{\mathrm{VL}} & M_{\mathrm{VI}} & M_{\mathrm{VR}} & M_{\mathrm{VV}} \end{array}\right]\left[\begin{array}{c} X_{L}\\ X_{I}\\ X_{R}\\ v \end{array}\right]=\left[\begin{array}{c} R_{A}\\ R_{I}\\ R_{B}\\ -q \end{array}\right]$$

where K is stiffness matrix and M is mass matrix, R and q represent the force and the net surface charge, respectively. X is particle displacement, the scalar v is the electric potential, the subscript L, I, R and V represent the position of freedom at the grid node.

If there is no external force, the quantities of $F_{L}$, $F_{I}$,$\;F_{R}$, imposing on the right, middle and left of grid node, respectively, are equal to zero, and the linear system equation can be expressed

(14) $$\left[\begin{array}{ccc} SYS_{LL} & SYS_{LI} & \begin{array}{cc} SYS_{LR} & SYS_{LV} \end{array}\\ SYS_{IL} & SYS_{II} & \begin{array}{cc} SYS_{IR} & SYS_{IV} \end{array}\\ \begin{array}{c} SYS_{RL}\\ SYS_{VL} \end{array} & \begin{array}{c} SYS_{RI}\\ SYS_{VI} \end{array} & \begin{array}{cc} \begin{array}{c} SYS_{RR}\\ SYS_{VR} \end{array} & \begin{array}{c} SYS_{RV}\\ SYS_{VV} \end{array} \end{array} \end{array}\right]\left[\begin{array}{c} X_{L}\\ X_{I}\\ \begin{array}{c} X_{R}\\ v \end{array} \end{array}\right]=\left[\begin{array}{c} 0\\ 0\\ \begin{array}{c} 0\\ -q \end{array} \end{array}\right]$$

On basis of this, we introduce MOR techniques based on FEM to further enhance its computation efficiency. MOR is not widely familiar to members of the SAW industry, which can deal efficiently with the large calculation under the condition of ensuring accuracy. The detailed scheme of the MOR technique is presented below: the internal DOFs $X_{I}$ is eliminated from the system Equation (14) by MOR technique, the system matrices *SYS* can be greatly reduced in dimensionality, and thus the Equation (15) only includes $X_{L}$, $X_{R}$ and the scalar v. In this case, *SYS*-matrices are transferred from RAM to the GPU to achieve acceleration due to GPUs have faster data processing capabilities than CPUs.

(15) $$\left[\begin{array}{ccc} SY{S^{\prime }}_{11} & SY{S^{\prime }}_{12} & SY{S^{\prime }}_{13}\\ SY{S^{\prime }}_{21} & SY{S^{\prime }}_{22} & SY{S^{\prime }}_{23}\\ SY{S^{\prime }}_{31} & SY{S^{\prime }}_{32} & SY{S^{\prime }}_{33} \end{array}\right]\left[\begin{array}{c} X_{L}\\ X_{R}\\ v \end{array}\right]=\left[\begin{array}{c} 0\\ 0\\ -q \end{array}\right]$$

It is noted that the reduced matrix Equation (15) is equivalent to the linear system Equation (14).

#### 2.2.3. MOR Based on Anti-Periodic Boundary Condition

Furthermore, considering the periodicity of the single-finger structure for the SAW resonator, we also further employing MOR based on the antiperiodic boundary conditions. The detailed process is presented that the antiperiodic boundary conditions ($X_{L}=-X_{R}$) is imposed on the left- and the right-hand edge of the block (shown in Figure 2a) for describing the repeating structure. Therefore, Equation (15) with reduction of the dimensionality can be reduced the dimensionality again by eliminating the DOFs $X_{R}$. Therefore, the linear system equations for the periodic structure only include the $X_{L}$ and v, as follows

(16) $$\left[\begin{array}{cc} SY{S^{\prime \prime }}_{11} & SY{S^{\prime \prime }}_{12}\\ SY{S^{\prime \prime }}_{21} & SY{S^{\prime \prime }}_{22} \end{array}\right]\left[\begin{array}{c} X_{L}\\ v \end{array}\right]=\left[\begin{array}{c} 0\\ -q \end{array}\right]$$

where

$$\begin{array}{c} SY{S^{\prime \prime }}_{11}=SY{S^{\prime }}_{11}+SY{S^{\prime }}_{21}-SY{S^{\prime }}_{12}-SY{S^{\prime }}_{22}\\ SY{S^{\prime \prime }}_{12}=SY{S^{\prime }}_{13}+SY{S^{\prime }}_{23}\\ SY{S^{\prime \prime }}_{21}=SY{S^{\prime }}_{31}-SY{S^{\prime }}_{32}\\ SY{S^{\prime \prime }}_{22}=SY{S^{\prime }}_{33} \end{array}$$

And finally, the expression of the currents flowing into the electrodes can be written as

(17) $$I=-i\omega \left[SY{S^{\prime \prime }}_{21}X_{L}+SY{S^{\prime \prime }}_{22}v\right]$$

## 3. Numerical and Experimental Results

Currently, mainstream commercial SAW devices use Lithium Tantalate (LT) and lithium niobte (LN) as piezoelectric materials. A series of SAW resonators fabricated on LT and LN based piezoelectric substrates, including IDT (Al)/42° YX LT structure, IDT (Cu)/42° YX LT/SiO_2_/Si and SiO_2_/IDT (Cu)/5° YX LN, are taken as examples for validating the accuracy and availability of the presented methodology. The fundamental material constants of the LT and LNsubstrate used for calculation are taken from the literature [48]. 

**Figure 3:**
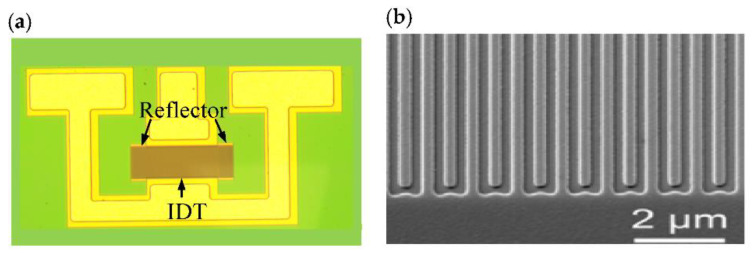
(**a**) Optical image and (**b**) SEM image for the fabricated one-port resonator.

Figure 3 shows the optical and scanning electron microscopy (SEM) images of the fabricated one-port SAW resonator. As shown in Figure 3b, the fabricated IDT pattern is well. All the fabricated resonators configured with IDTs consisting of 100 pairs of fingers and 40 grating reflectors placed on both sides of the IDTs, and aperture length of 40 IDT period *p* (*p* = λ, where λ is the SAW wavelength). The measured Y11 of SAW resonators is characterized by an Aglient E5071C vector network analyzer (Keysight Technologies, Roseville, CA, USA) with a vector network analyzer and ground–signal–ground (GSG) probes.

### 3.1. LT-Based SAW Resonator

**Figure 4:**
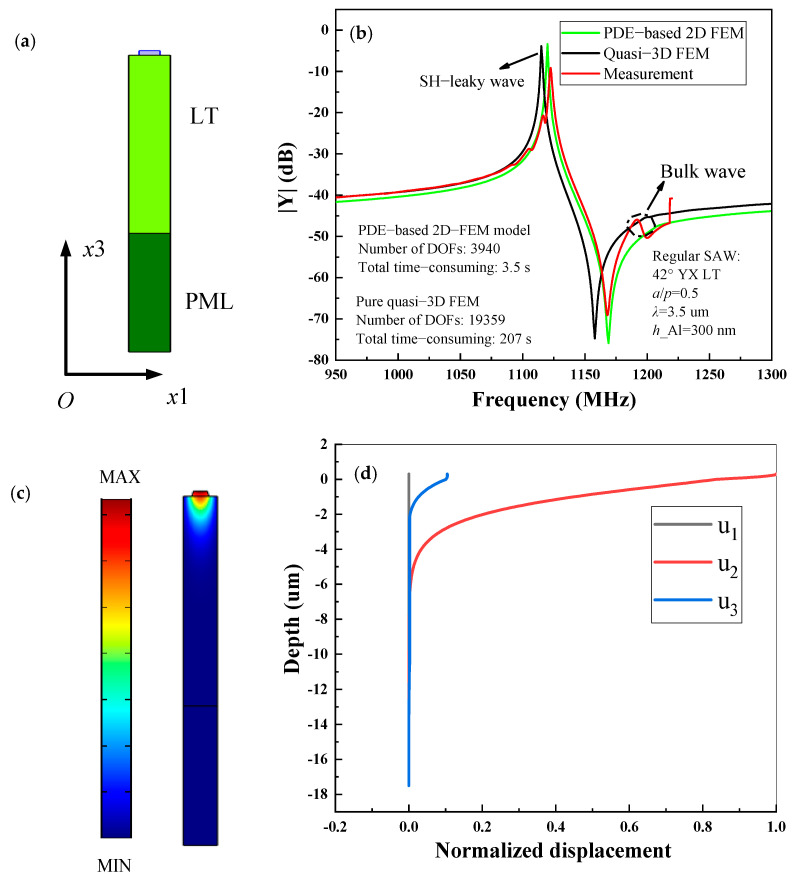
Regular SAW resonator on Al/42° YX LT structure, (**a**) partial differential equations (PDE)-based 2D-FEM model, (**b**) *Y*_11_ curves for simulation and experiment, (**c**) the mode shape at the resonant frequency, (**d**) displacement field distribution versus normalized depth for the SH-leaky SAW at the resonant frequency.

Figure 4a shows the single-finger 2D-FEM model of regular SAW resonator on Al/42° YX cut LT structure. In this case, the IDT period *p* = 3.5 µm, the thickness of Al electrode *h* = 300 nm, and the thickness of LN is 3*p*. In the following calculation, the thickness of the bottom substrates is always set as 3*p*, and the thickness of the PML is set as 2*p*. The input admittance *Y*_11_ curves of SAW resonators are presented for comparison in Figure 4b. As can be seen clearly, the simulated result based on the PDE-based 2D-FEM model using COMSOL code fits better with the experiment than that of pure quasi-3D FEM. This is mainly because the propagation loss, dielectric loss, electrode resistance loss and electrode shape are fully considered into the proposed model in our work by modifying PDEs themselves. As can be seen, the measured curve exists the bulk wave at ~1.2 GHz due to the wave reflection among the finger of practical SAW resonator with finite-length, which does not affect the coupling coefficient and quality factor *Q* of the main SAW mode we were concerned with. In addition, the simulation speed of the PDE-based 2D-FEM method is about 0.01 s for each frequency point and its total time cost is 3.5 s in the frequency range from 950 MHz to 1300 MHz, while the pure quasi-3D FEM model requires about 0.59 s for each frequency point and its total time cost is 207 s due to the amounts of DOFs. It is noted that the above calculations are under the same frequency interval of 1 MHz. Thus, the periodic analysis is still a powerful simulation tool for researchers to analyze the performance of the SAW mode we were concerned with, and important SAW properties including the phase velocity, coupling coefficient, and relative bandwidth, etc., can be obtainable from the calculated Y11.

Figure 4c,d show the calculated mode shape and the displacement field distributions entirely composed of three partial components at the resonance frequency, respectively. It is quite clear that the acoustic energy is concentrated near the surface, and the displacements along x direction (*u*_1_) are close to zero while the horizontal (*u*_2_) and vertical shear components (*u*_3_) are dominant, and the horizontal shear (SH) components (*u*_2_) is significantly larger than vertical shear components (*u*_3_). This means the main mode is SH-leaky SAW, namely, acoustic waves are mainly horizontally polarized and radiate into the body at an angle. 

Furthermore, the SAW resonator fabricated on IHP-type hetero layered structure Cu/42° YX LT/SiO_2_/Si is also calculated. Figure 4a shows the single-finger 2D-FEM model of the IHP-type SAW resonator used for simulation. In this case, the IDT period *p* = 1.8 µm (*λ* = 2 ∗ *p*), and the thickness of Cu electrode *h* = 160 nm. The thickness of LT and SiO_2_ are 600 nm and 500 nm, respectively. Figure 4b shows the input admittance *Y*_11_ curve of SAW devices for measurement and calculation by PDE-based 2D-FEM model and pure quasi-3D FEM, respectively. As shown, the results are similar to that of the normal SAW on Al/42° YX LT case. The simulated results are in fairly well agreement with that of the experiment for the main wave mode we were concerned with, while the bulk spurious mode is missed. The simulation speed of the PDE-based 2D-FEM method is about 0.012 s for each frequency point and its total time cost is 9.6 s in the frequency range from 1550 MHz to 2350 MHz, but the pure quasi-3D FEM model requires about 0.51 s for each frequency point and its total time cost is 410 s.

**Figure 5:**
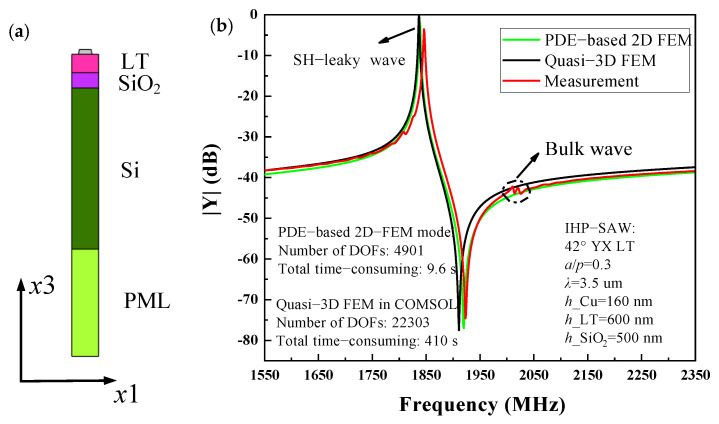

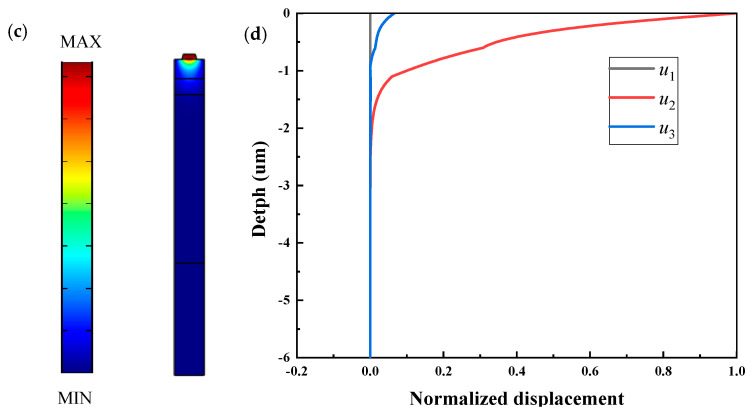
IHPSAW resonator on Cu/ 42° YX LT/SiO_2_/Si structure, (**a**) PDE-based 2D-FEM model, (**b**) *Y*_11_ curves for simulation and experiment, (**c**) the mode shape at the resonant frequency, (**d**) displacement field distribution versus normalized depth for the SH-leaky SAW at the resonant frequency.

Figure 5c,d show the calculated mode shape and the displacement field distributions entirely composed of three partial components at the resonance frequency in IHP-type SAW resonator, respectively. The displacements along x direction (*u*_1_) are close to zero while the horizontal (*u*_2_) and vertical shear components (*u*_3_) are dominant, and the horizontal shear components (*u*_2_) are significantly larger than vertical shear components (*u*_3_). Compared to normal SAW resonators on LT, it is quite clear that more acoustic energy is concentrated near the surface and thus offers enhanced performance.

### 3.2. LN-Based SAW Resonator

**Figure 6:**
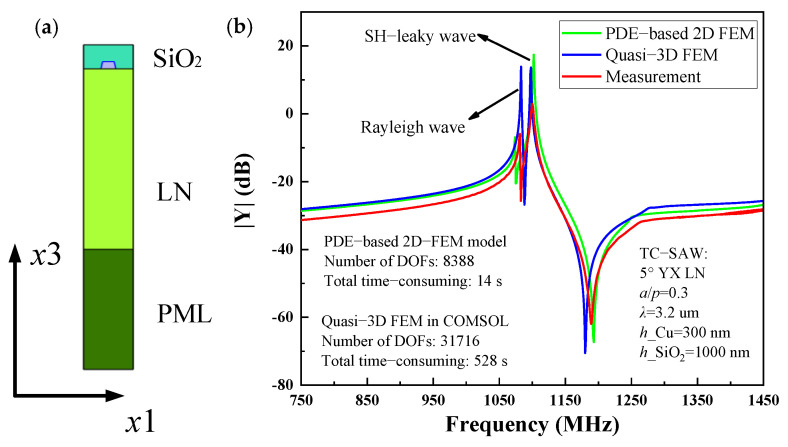

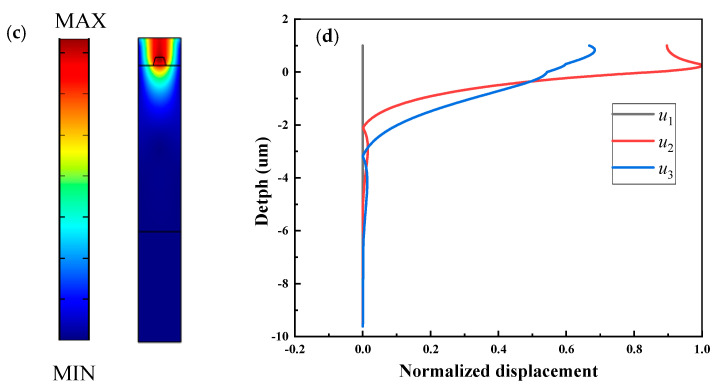
Temperature-compensated SAW (TC-SAW) resonator on SiO_2_/IDT/5° YX-LN structure, (**a**) PDE-based 2D-FEM model, (**b**) *Y*_11_ curves for simulation and experiment, (**c**) the mode shape at the resonant frequency, (**d**) displacement field distribution versus normalized depth for the SH-leaky SAW at the resonant frequency.

Temperature-compensated (TC) SAW resonators are more preferable for practical application because of their good temperature stability. In this case, the typical TC-SAW resonator fabricated on SiO_2_/IDT/5° YX-LN structure is investigated. Figure 6a shows the single-finger 2D-FEM model of TC-SAW resonator used for simulation. The IDT period *p* = 4 µm, the thickness of Al electrode *h* = 380 nm, and the thickness SiO_2_ is 600 nm. Figure 6b shows the simulated and measured Y11 of the TC-SAW resonator for comparison. One can clearly see that the simulated result agrees well with measurement based on the PDE-based 2D-FEM model using COMSOL code. It is noted that the spurious wave of Rayleigh wave at ~1100 MHz is also characterized. Compared to pure quasi-3D FEM, the characteristics of the spurious wave are more accurate due to more practical factors taken into account. In addition, the total time cost of the proposed method is 14 s in the frequency range from 750 MHz to 1450 MHz, but the pure quasi-3D FEM model requires a total time cost of 528 s due to the amounts of DOFs.

Figure 6c,d show the calculated mode shape and the displacement field distributions entirely composed of three partial components at the resonance frequency in the TC-SAW resonator, respectively. The displacements along x direction (*u*_1_) are close to zero while the horizontal (*u*_2_) and vertical shear components (*u*_3_) are dominant. It is interesting that the horizontal shear components (*u*_2_) and vertical shear components (*u*_3_) are relatively large. The reason is mainly that the structure of the TC-SAW device is configured with an embedded electrode, the displacement and stress boundary conditions on piezoelectric interface change with not only electrode but also SiO_2_, which results in more intense coupling of the partial acoustic surface waves (*u*_1_, *u*_2_, *u*_3_). In addition, although most acoustic energy is concentrated near the surface, some still partly radiates into the substrate. Therefore, for the TC-SAW resonator, a very important question is how to eliminate spurious waves.

### 3.3. Results Comparison and Discussions

For quantitative analysis, the key performance parameters of those SAW resonators including electromechanical coupling coefficient (*K*^2^) and quality factor (*Q*) are evaluated based on the calculated frequency behavior. Its detailed formula is reported in our previous work [49]. Table 1 presents the key performance parameters of those SAW resonators. As shown, the results of the periodic analysis compared fairly well with the experimental results of the actual SAW devices including the regular SAW resonator, IHP-SAW resonator and TC-SAW resonator. The relative errors for *K*^2^ and *Q* are less than 4% and 3%, respectively. As the proposed PDE-based FEM model fully takes account of the propagation loss, dielectric loss, electrode resistance loss and electrode shape, the differences between the simulated and measured results are mainly due to practical fabrication error and the ignored self-inductance and mutual inductance of the busbars and electrodes. Table 2 shows the comparison in calculation speeds between the proposed PDE-based 2D-FEM model and the previously reported quasi-3D FEM model. It is seen that the proposed model gives rise to largely reduced DOFs, thus achieving 38–60 times speedup compared to the quasi-3D model for the same frequency interval and configuration.

The above quantitative comparative results demonstrate the effectiveness and accuracy of the proposed model. This means the periodic analysis is still a relatively powerful simulation tool for researchers to analyze the performance of the SAW mode we were concerned with, and important SAW properties including *Q* and *K*^2^ as well as phase velocity and relative bandwidth, etc., can be estimated.

## 4. Conclusions

This paper presents a reduced PDE-based 2D-FEM model using COMSOL code for the periodic analysis of the SAW resonator. By performing MOR techniques based on FEM and periodic boundary conditions, the dimensionality of the final system equation for characterizing the SAW resonator can be reduced largely, therefore, a dimensionally reduced PDE model without decreasing the accuracy of computations can be achieved. Furthermore, the proposed PDE-based 2D-FEM model is performed on examples of three different mainstream commercial SAW devices, including the regular SAW, IHP-SAW and TC-SAW resonators. For validity, the results of the periodic analysis are compared with the experimental results of the actual resonators. The calculated input admittance *Y*_11_ curves of the SAW resonators agree fairly well with that of the experimental results, which demonstrates the properties of the proposed methodology and prove its effectiveness and accuracy. 

There are advantages and disadvantages to using a periodic structure analysis. Although the behavior of the periodic structure may not resemble that of the whole resonator, for example, bulk spurious modes may be missed, it can offer useful and enough information of the concerned main SAW mode. Moreover, the proposed dimensionally-reduced PDE model using COMSOL code is still an efficient and general simulation tool for diverse SAW resonator analysis and design, especially for the development of new-type SAW devices configured with complex structures. 

## Figures and Tables

**Table 1:** The compared key performance parameters of SAW resonator for simulation and experiment, S donates the simulation results calculated by the proposed PDE-based 2D FEM model, M donates the measurement experimental results.

Parameters	Normal SAW			IHP SAW			TC SAW		
	S	M	Error	S	M	Error	S	M	Error
*K* ^2^	9.64%	9.31%	3.42%	10.53%	10.28%	2.37%	18.82%	18.51%	1.65%
*Q*	549.18	551	0.33%	1011	986.8	2.39%	976.1	947.96	2.88%

**Table 2:** Comparison of different methods for SAW devices simulation, T1 donates calculation speed per frequency point, T2 donates total calculation time in the frequency range, and the frequency interval remains as 1 MHz.

FEM Model	Normal SAW			IHP SAW			TC SAW		
	DOFs	T1 (s)	T2(s) (950–1300 MHz)	DOFs	T1 (s)	T2(s) (1550–2350 MHz)	DOFs	T1 (s)	T2 (s) (750–1450 MHz)
PDE	3940	0.01	3.5	4901	0.012	9.6	8388	0.02	14
Quasi-3D	19359	0.59	207	22303	0.51	410	31716	0.75	528
